# Associated lesions in posterior wall acetabular fractures: not a valid predictor of failure

**DOI:** 10.1007/s10195-013-0247-x

**Published:** 2013-06-04

**Authors:** Lukas Daniel Iselin, Peter Wahl, Patrick Studer, Jacob T. Munro, Emanuel Gautier

**Affiliations:** 1 Department of Traumatology, University Hospital Basel, 4031 Basel, Switzerland; 2 Department of Orthopaedic Surgery and Traumatology, Kantonsspital Fribourg, 1700 Fribourg, Switzerland; 3 University of Auckland, Auckland, New Zealand

**Keywords:** Acetabulum, Posterior wall acetabular fractures, Associated lesion, Outcome, Prognosis

## Abstract

**Background:**

The general outcome of posterior wall acetabular fractures is still the source of discussion. Posterior wall fractures are recognized throughout the literature as being difficult to treat. The aim of the present study was to analyze in our own patients the relevance of the classical prognostic criteria for the outcome of isolated posterior wall fractures and those with associated lesions.

**Materials and methods:**

A prospective cohort of 33 consecutive patients treated operatively between 1996 and 2006 in a single level 1 trauma center for a posterior wall fracture of the acetabulum was analyzed retrospectively. Included were posterior wall acetabular fractures or associated posterior wall fractures, such as the combinations of posterior column with posterior wall, transverse with posterior wall, or T-shaped fracture with posterior wall fracture. Outcome measurement of the postoperative survival of the hip joints until the primary outcome reoperation (total hip replacement or fusion) and secondary outcome diagnosis of symptomatic osteoarthritis were performed.

**Results:**

Twenty-six of the 33 patients with posterior wall fractures also had a dislocated joint. Twelve had isolated and 21 associated fractures. Six patients were reoperated with a THA (four patients within 2 years and one after 10 years), and one arthrodesis was done to treat a hematogenous septic arthritis in a degenerative hip joint. Secondary arthritis was observed in 10 patients.

**Conclusions:**

No difference was found between the outcome in cases of isolated posterior wall acetabular fracture and the outcome in those with associated lesions. The classical prognostic criteria were not found to be relevant to the outcome for our group.

## Introduction

Acetabular fractures are frequently associated with high-impact trauma, especially road traffic accidents. These often involve young, active people, so precise diagnosis and a well-executed treatment plan are vital in order to achieve a good functional result that is durable over the long term.

Scientific discussion has focused on the classification of the fracture pattern and is based on the pioneering works of Merle d’Aubigné, Judet, and Letournel [1–3]. Letournel and Matta et al. [4–6] introduced the importance of the fracture classification in determining prognosis. Mears et al. [7] showed that parameters such as marginal impaction, lesion of the femoral head and femoral neck fractures, severe obesity, and especially older age should be taken into consideration in clinical practice in order to determine the severity and the prognosis of acetabular fractures.

Fractures of the posterior wall of the acetabulum may be isolated or associated with injuries to other local anatomical structures. These associated injuries might be acetabular, such as posterior column, transverse, or T-configuration fractures, or intra-articular injuries such as a multi-fragmentary fracture pattern, marginal impaction, intra-articular fragments, and lesions of the femoral head [7]. The aim of this study was to analyze the prognostic value of the presence of associated fracture patterns and the prognostic parameters usually used for the functional and radiologic outcome of acetabular posterior wall fractures in our patients.

## Materials and methods

We conducted a study of all patients treated surgically for a posterior wall acetabular fracture between January 1996 and November 2006 at our institution. While the study was retrospective, the relevant demographic, operative, and follow-up data for all patients treated in our institution for acetabular fractures were collected prospectively in an Excel^®^-based (Microsoft, Redmond, WA, USA) database. Identification of patients who suffered a posterior wall acetabular fracture was achieved through a keyword search.

The study was authorized by the internal review board and was performed in accordance with the ethical standards of the 1964 Declaration of Helsinki as revised in 2000. All patients gave their informed consent prior to being included in the study.

The database search allowed the identification of 33 consecutive patients who had suffered a posterior wall acetabular fracture. They were all operated on by the senior author. Operative treatment was chosen in cases with displacement >2 mm, presence of intra-articular fragments, and/or lack of containment of the femoral head. Clinical and radiological follow-up examinations were done systematically at 6 weeks, 3 months, and 1 year postoperatively, and at other time points (depending on the evolution).

Postoperatively, all patients underwent the same management, with early mobilization and partial weight bearing of 15 kg during the first 6 weeks, which was usually increased to total weight-bearing after 3 months and radiologic fracture consolidation. Hip flexion was limited to 70° for 6 weeks. During that time, no active or passive flexion of the leg was allowed when the patient was lying on their back. In cases with a trochanteric osteotomy, active hip abduction was restricted for 6 weeks.

The following parameters, which are considered to be potentially significant prognostic indicators of the outcome, were collected: type of acetabular fracture according to the classification of Letournel [1, 4], joint dislocation at admission, presence of intra-articular fragments, marginal impaction, femoral head lesion, and number of main fragments. We used the 1986 modified Merle d’Aubigné Score and the well known Harris Hip Score to monitor the postoperative evolution of every patient [1, 8]. To facilitate comparisons of Merle d’Aubigné scores, all 3 parameters were added up to give a total maximum score of 18 points (as commonly done, even though the original score evaluates every parameter separately) [1].

The three X-rays (AP pelvis, ala and obturator views) taken immediately after surgery were reinterpreted by the first author and the director of the study. Radiological assessment of the surgical reposition and fixation of the fractures was done according to Matta’s criteria, which are used to document and grade the maximal dislocation on the three standard X-rays [5]. Three degrees of displacement are described, ranging from anatomical (under 1 mm) to bad (over 3 mm). Heterotopic ossification was classified according to Brooker [9]. Quantification of the posttraumatic radiologic changes utilized the arthritis classification according to Kellgren and Lawrence, and necrosis of the femoral head was quantified according to ARCO (Association Research Circulation Osseous) [10, 11].

Demographic data, fracture classification and concomitant injuries, and operative data, separated into data relating to the outcome of reoperation and that relating to the outcome of diagnosis of symptomatic post-traumatic osteoarthritis of the hip joint, are indicated in Table 1.

**Table 1:** Overall demographic and fracture descriptive data

Variable	Results
Number of cases *n*	33
Average age in years (SD, range)	34 (17, 10–70)
Sex *n* male:*n* female (%:%)	28:5 (85 %:15 %)
Side *n* right:*n* left (%:%)	17:16 (52 %:48 %)
Cause *n* (%)	
Car accident	25 (76 %)
Motorbike accident	4 (12 %)
Fall, sports accident	4 (12 %)
Fracture type *n* isol. PW: *n* associated PW (%:%)	12:21 (36 %:64 %)
Joint dislocation at admission *n* (%)	28 (85 %)
Multiple fragments *n* (%)	25 (76 %)
Marginal impaction *n* (%)	13 (39 %)
Intra-articular fracture *n* (%)	13 (39 %)
Femoral head lesion *n* (%)	18 (55 %)
Traumatic nerve palsy *n* (%)	5 (15 %)
Average delay to injury-related surgery in days (SD, range)	8 (7.6, 1–40)
Average surgery time in minutes (SD, range)	202 (49, 120–300)
Average follow-up period in months (SD, range)	55 (34, 12–135)
Secondary osteoarthritis *n* (%)	9 (27 %)
Reoperation *n* (%)	
Total hip replacement	5 (15 %)
Arthrodesis	1 (3 %)

*PW* posterior wall

The delay between initial trauma and definitive surgical treatment of the acetabular fracture was on average 7 days (range: 1–22 days). Twenty-six patients were operated on using a posterior approach according to Kocher-Langenbeck with a trochanteric flip osteotomy; an isolated Kocher-Langenbeck approach was used in 5 patients, while an ilio-inguinal approach in combination with the Kocher-Langenbeck with a trochanteric flip osteotomy was performed in 2 patients.

Endpoints were the diagnosis of post-traumatic symptomatic osteoarthritis and reoperation (for total hip replacement or arthrodesis), which were both recorded for the survival analysis. Follow-up was censored when one of these endpoints appeared, or on the date of the last follow-up exam. Clinical and radiological follow-up was completed at 6 and 12 weeks postoperatively and at 1 year after surgery. All patients were seen or contacted by mail or a phone call once a year. The mean postoperative follow-up was 4.5 years. No patient died within the observation period.

Statistical analysis was done using both MedCalc^®^ version 10.4 (MedCalc Software, Mariakerke, Belgium) and SPSS^®^ Statistics version 12.0 (SPSS Inc., Chicago, IL, USA). Depending on the characteristics of the data, comparisons were performed using Student’s *t* test, Fisher’s exact test, or the chi-square test. Survival analysis was done using the Kaplan–Meier method, with the Breslow test used for inter-group comparisons. A statistical significant difference was accepted for *p*-values of <0.05. The sample size estimation/power analysis was done using the online calculator available at http://www.stat.ubc.ca/~rollin/.

## Results

**Fig. 1:**
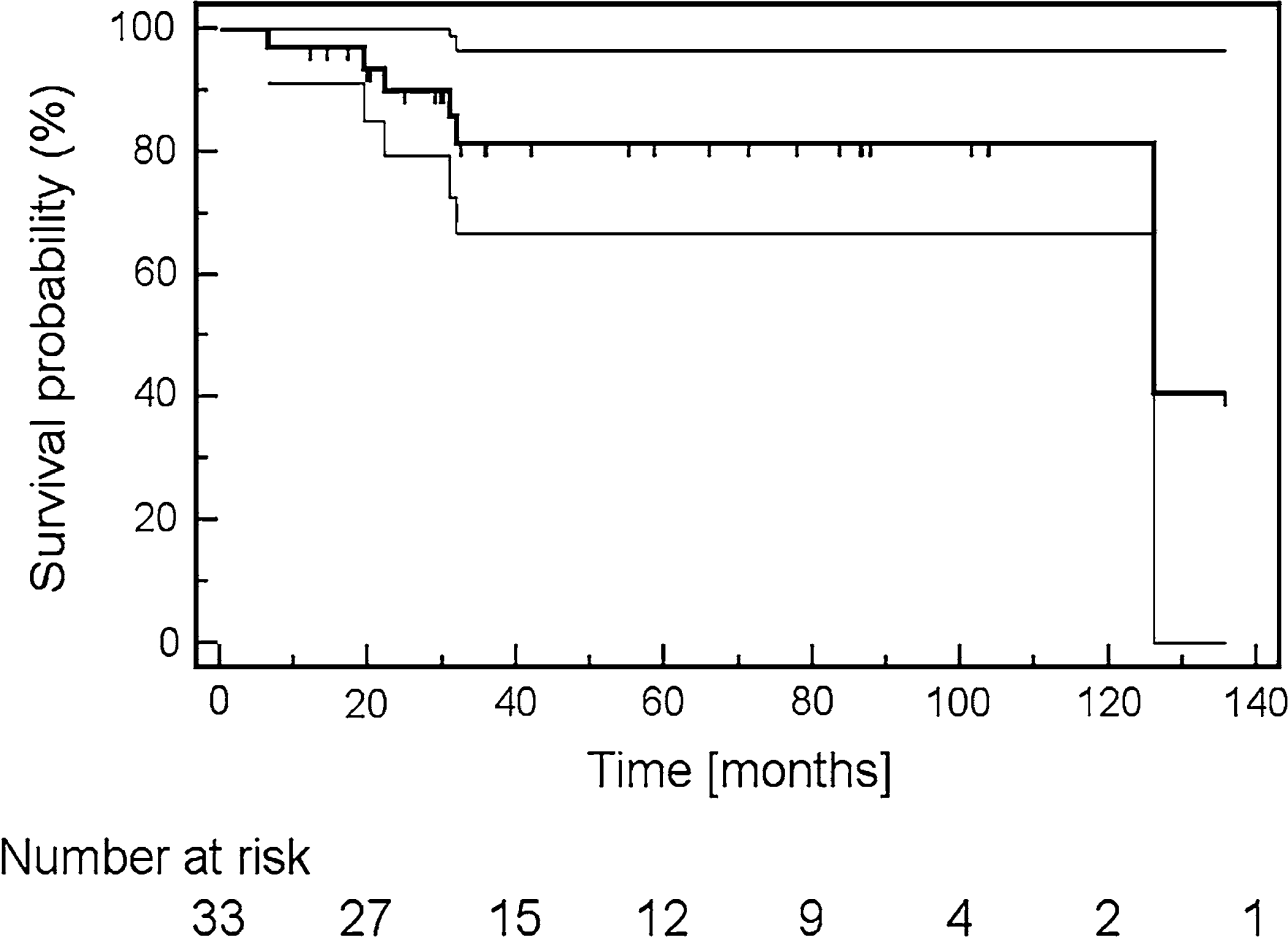
Kaplan–Meier survival curve with reoperation—be it total hip replacement or hip arthrodesis—as the endpoint after acetabular fracture affecting the posterior wall.*Thin lines* delimit the 95 % confidence interval. *Tick marks* indicate cases that were censored as dictated by the availability of follow-up

The primary outcome measure assessment revealed that reoperation was necessary in 6 patients. Total hip replacement was performed in 4 patients within 2 years and in 1 after 10 years, and 1 arthrodesis was done in 1 patient after 32 months. The latter was a case of hematogenous septic arthritis on a severely degenerated hip joint in an iv-drug abuser. The Kaplan–Meier survival curve with reoperation as the endpoint is shown in Fig. 1. Among the studied group of patients, 10 were diagnosed with a symptomatic post-traumatic osteoarthritis of the hip joint. Except in 1 case where the diagnosis was made after 3 years and another case where it was made after 10 years, the diagnosis was always made within 2 years following the operation.

As summarized in Table 2, the statistical evaluation was not able to show that the associated lesions that are usually considered have predictive value regarding the outcome of the development of post-traumatic osteoarthritis or the need for hip joint replacement or fusion. Excluding this case of arthrodesis from the analysis did not change the results significantly.

**Table 2:** Patient and fracture characteristics by outcome

Variable	Not reoperated	Reoperated	Statistics	No osteoarthritis	Osteoarthritis	Statistics
Number of cases *n* (%)	27 (82 %)	6 (18 %)		23 (70 %)	10 (30 %)	
Average age in years (SD, range)	35 (15, 13–69)	49 (21, 27–75)	*p* = 0.08 (Student)	37 (16, 13–69)	40 (20, 20–75)	*p* = 0.6 (Student)
Sex *n* male:*n* female (%: %)	23:4 (85 %:15 %)	5:1 (83 %:17 %)	*p* = 1.0 (Fisher)	20:3 (87 %:13 %)	8:2 (80 %:20 %)	*p* = 0.6 (Fisher)
Side *n* right:*n* left (%: %)	13:14 (48 %:52 %)	3:3 (50 %:50 %)	*p* = 1.0 (Fisher)	11:12 (48 %:52 %)	5:5 (50 %:50 %)	*p* = 1.0 (Fisher)
Cause, *n* (%)			*p* = 0.3 (*χ*^2^)			*p* = 0.15 (*χ*^2^)
Car accident	19 (70 %)	6 (100 %)		16 (70 %)	9 (90 %)	
Motorbike accident	4 (15 %)	0 (0 %)		3 (13 %)	1 (10 %)	
Fall, sports accident	4 (15 %)	0 (0 %)		4 (17 %)		
Fracture type						
* n* isol. PW: *n* assoc. PW (%:%)	11:16 (41 %:59 %)	1:5 (17 %:83 %)	*p* = 0.4 (Fisher)	11:12 (48 %:52 %)	1:9 (10 %:90 %)	*p* = 0.054 (Fisher)
Joint dislocation at admission *n* (%)	22 (81 %)	6 (100 %)	*p* = 0.6 (Fisher)	18:5 (78 %:22 %)	10:0 (100 %:0 %)	*p* = 0.3 (Fisher)
Multiple fragments *n* (%)	19 (70 %)	6 (100 %)	*p* = 0.3 (Fisher)	15 (65 %)	10 (100 %)	*p* = 0.07 (Fisher)
Marginal impaction *n* (%)	10 (37 %)	3 (50 %)	*p* = 0.7 (Fisher)	9 (39 %)	4 (40 %)	*p* = 1.0 (Fisher)
Intra-articular fracture *n* (%)	10 (37 %)	3 (50 %)	*p* = 0.7 (Fisher)	7 (30 %)	6 (60 %)	*p* = 0.14 (Fisher)
Femoral head lesion *n* (%)	14 (52 %)	4 (67 %)	*p* = 0.7 (Fisher)	11 (48 %)	7 (70 %)	*p* = 0.3 (Fisher)
Traumatic nerve palsy *n* (%)	5 (19 %)	0 (0 %)	*p* = 0.6 (Fisher)	5 (22 %)	0 (0 %)	*p* = 0.3 (Fisher)
Average delay to injury surgery in days (SD, range)	8 (8, 1–40)	7 (4, 3–12)	*p* = 0.6 (Student)	7 (5, 1–18)	11 (12, 3–40)	*p* = 0.089 (Student)
Average surgery time in httpminutes (SD, range)	193 (47, 120–270)	238 (42, 180–300)	*p* = 0.039 (Student)	185 (46, 120–270)	239 (34, 180–300)	*p* = 0.003 (Student)
Average follow-up in months (SD, range)	54 (33, 12–135)	59 (39, 25–133)	*p* = 0.8 (Student)	56 (35, 12–135)	53 (33, 25–133)	*p* = 0.8 (Student)

*Fisher*Fisher’s exact test, *Student*Student’s *t* test,*χ*^2^ chi square test

The operation time was on average 202 min for all patients studied, but about 40 min longer in the patients who later required reoperation. While the difference appeared to be statistically significant for both outcomes, operation time values showed a significant overlap between both groups. No early complications such as wound infection, hematoma, or neural injuries related to surgery were seen.

The reduction of the fractures was highly satisfactory in all patients, with <2-mm gaps or steps. Heterotopic ossification of Brooker grade I was observed in 5 patients, grade II in another 6 patients, but no higher grade was seen. For the whole study group, the final median total Merle d’Aubigné Score was 17/18 (range: 11–18), with only 3 cases with a score below 14, among whom two had a score of only 11 points, corresponding to good results in 90 % of the cases. The median Harris Hip Score at final follow-up was 96/100 (range: 66–100). The score was above 80/100 in all patients, including 1 patient whose score had been at 66/100 3 months postoperatively. Excluding the patients who were reoperated, the final median Merle d’Aubigné Score and Harris Hip Score remained unchanged.

## Discussion

In this study of 33 patients who had suffered fractures of the acetabulum affecting the posterior wall, 10 (30 %) developed symptomatic osteoarthritis, and 6 (18 %) of these required reoperation for total hip replacement or hip arthrodesis. The one case of arthrodesis was an iv-drug abuser who had developed hematogenous septic arthritis on a severely degenerated hip, which was not, however, symptomatic to a point that it would otherwise have required an operation. When this patient was excluded, the reoperation rate remained at 15 %. This is quite comparable to the results obtained from other series published in the literature [7, 12–20]. It is worth noting that nearly all reoperations were done within the first 2 years postoperatively, as illustrated in Fig. 1. The need for an arthroplasty thus appears rather early after internal fixation, and not over the long term.

None of the examined fracture patterns had a statistically significant association with the development of osteoarthritis and the requirement for reoperation. This could certainly be due to the small number of patients analyzed in this study. On the other hand, it also might be that these parameters are not decisive regarding outcome after posterior wall acetabular fracture. This is also illustrated by the fact that the importance of certain parameters, such as marginal impaction, could not be documented uniformly in all studies [12–15].

Interestingly, increased surgery time was associated in this study with the appearance of a symptomatic post-traumatic osteoarthritis and the requirement for reoperation. As all operations were done by the same surgeon, this might be an indicator of the difficulty of the operation—a more sensitive one than fracture pattern descriptors. The time values overlapped too much for this parameter to be of clinical utility. The effect size of 0.92 means that the outcome could be deduced correctly from the operation time only 2 out of 3 times. Similarly, it had been expected that associated posterior wall fractures, which are more complicated than isolated posterior wall fractures, would show a worse outcome, but this did not reach statistical significance in our series.

A trochanteric flip osteotomy was used in a rather large proportion of the cases in this series. While it might be mandatory in only a small proportion of cases, it is our conviction that surgeons should not refrain from using this extension of the approach to gain adequate exposure and to be able to dislocate the hip joint in order to verify intra-articular reduction and adequacy of screw placement. The described series, however, is not able to provide evidence to support this.

Limitations of our study are certainly the restrospective nature of the register study and its limited statistical power due to the rather small number of cases available. Acetabular fractures remain rare and difficult to treat. Details from this study may, however, be included in larger reviews that could refine the prognostic criteria for this kind of fracture. While reoperation is a hard outcome, a potential bias should not be forgotten. Even if patients get symptomatic from a post-traumatic osteoarthritis, young age might refrain from reoperation. For this reason, the appearance of the diagnosis of symptomatic post-traumatic osteoarthritis was also analyzed, even though it was highly subjective and thus a rather weak parameter.

In order to estimate how many cases would be neeeded to be able to detect a statistically significant association of the examined parameters with the outcome reoperation, sample size estimation can be done as for a case–control study, with patients without reoperation being considered controls. A one-sided power analysis might be considered, as it is expected that the prevalence of the risk factors should be higher in the group of re-operated patients. The relative risk of all observed parameters was, however, below 1.5. Considering the common values for type I error risk (5 %) and type II error risk (20 %), and an overall risk factor prevalence of 75 % as approximated by finding the mean of the values observed, it would require the reoperation of an estimated 450 cases to detect a statistically significant association. If the relative risk could be increased to optimistic 2, 169 cases would still be required. The power of case–control studies can certainly be optimized by using 2–4 times more controls than cases. However, as only 20–25 % of the patients who suffer such fractures require later reoperation, the number of patients required for such a study far exceeds the numbers a single institution can provide.

Large registers are probably the only way to answer the issue of prognostic factors in this type of fracture. Hopefully, the data provided here will at least be detailed enough to be included in any review to come.
